# Community-wide analysis of microbial genome sequence signatures

**DOI:** 10.1186/gb-2009-10-8-r85

**Published:** 2009-08-21

**Authors:** Gregory J Dick, Anders F Andersson, Brett J Baker, Sheri L Simmons, Brian C Thomas, A Pepper Yelton, Jillian F Banfield

**Affiliations:** 1 Department of Earth and Planetary Science, University of California, 307 McCone Hall, Berkeley, CA 94720, USA; 2 Department of Environmental Science, Policy, and Management, University of California, Hilgard Hall, Berkeley, CA 94720, USA; 3 Current address: Department of Geological Sciences, University of Michigan, 1100 N. University Ave, Ann Arbor, MI 48109-1005, USA; 4 Current address: Evolutionary Biology Centre, Department of Limnology, Uppsala University, Norbyv. 18 D, SE-75236, Uppsala, Sweden; 5 Current address: Department of Bacteriology, Swedish Institute for Infectious Disease Control, Nobels väg 18 SE-17182 Solna, Sweden

## Abstract

**Background:**

Analyses of DNA sequences from cultivated microorganisms have revealed genome-wide, taxa-specific nucleotide compositional characteristics, referred to as genome signatures. These signatures have far-reaching implications for understanding genome evolution and potential application in classification of metagenomic sequence fragments. However, little is known regarding the distribution of genome signatures in natural microbial communities or the extent to which environmental factors shape them.

**Results:**

We analyzed metagenomic sequence data from two acidophilic biofilm communities, including composite genomes reconstructed for nine archaea, three bacteria, and numerous associated viruses, as well as thousands of unassigned fragments from strain variants and low-abundance organisms. Genome signatures, in the form of tetranucleotide frequencies analyzed by emergent self-organizing maps, segregated sequences from all known populations sharing < 50 to 60% average amino acid identity and revealed previously unknown genomic clusters corresponding to low-abundance organisms and a putative plasmid. Signatures were pervasive genome-wide. Clusters were resolved because intra-genome differences resulting from translational selection or protein adaptation to the intracellular (pH ~5) versus extracellular (pH ~1) environment were small relative to inter-genome differences. We found that these genome signatures stem from multiple influences but are primarily manifested through codon composition, which we propose is the result of genome-specific mutational biases.

**Conclusions:**

An important conclusion is that shared environmental pressures and interactions among coevolving organisms do not obscure genome signatures in acid mine drainage communities. Thus, genome signatures can be used to assign sequence fragments to populations, an essential prerequisite if metagenomics is to provide ecological and biochemical insights into the functioning of microbial communities.

## Abstract 2

Genome signatures are used to identify and cluster sequences de novo from an acid biofilm microbial community metagenomic dataset, revealing information about the low-abundance community members.

## Background

The age of genomics has opened up new perspectives on the natural microbial world, offering insights into organisms that drive geochemical cycles and are critical to human and environmental health. The prevalence of horizontal gene transfer, recombination, and population-level genomic diversity underscores the dynamic nature of bacterial and archaeal genomes and demands reconsideration of fundamental issues such as microbial taxonomy [1,2] and the concept of microbial species [3,4]. Application of genomics to uncultivated assemblages of microorganisms in natural environments ('metagenomics' or 'community genomics') has provided a new window into *in situ *microbial diversity and function [5-7]. To date, community genomics has revealed the form and extent of recombination and heterogeneity in gene content [8-11], elucidated virus-host interactions [12], redefined the extent of genetic and biochemical diversity in the oceans [13-15], uncovered new metabolic capabilities [16-19] and taxonomic groups [20], and shown how functions are distributed across environmental gradients [21].

An important approach to study evolutionary and ecological processes, pioneered by Karlin and others [22], is the analysis of nucleotide compositional characteristics of genomes. The simplest and most widely used measure of nucleotide composition, the abundance of guanine plus cytosine (%GC), is shaped by multiple factors encompassing both neutral and selective processes. Neutral factors include intrinsic properties of the replication, repair, and recombination machinery that result in mutational biases [23,24]. Selective processes encompass both internal (for example, translation machinery) and external influences such as physical (temperature, pressure), chemical (salinity, pH) and ecological factors (competition for metabolic resources [25] and niche complexity [26]). Although the relative importance of these factors remains uncertain [27], it is clear that %GC varies widely between species but is relatively constant within species. Thus, %GC has been used to trace origins of DNA fragments within genomes [28] and to assign fragmentary metagenomic sequences to candidate organisms [16]. Such inferences must be made with caution: %GC simplifies nucleotide composition down to a single parameter with known limitations for investigating genome dynamics [29].

Oligonucleotide frequencies capture species-specific characteristics of nucleotide composition more effectively than %GC [30]. Analyses of genome sequences from cultivated organisms have shown that the frequency at which oligonucleotides occur is unique between species while being conserved genome-wide within species [22,30-34]. Taken together, the frequency of all oligonucleotides of a given length defines the 'genome signature' (for example, the frequency of all possible 256 tetranucleotides). Sequence signatures are evident in oligonucleotides ranging from di- (two-mers) to octanucleotides (eight-mers). While the specificity of genome signatures increases with oligonucleotide length [35], the number of possible oligomers increases exponentially with oligomer length, so signatures based on longer oligomers require calculations over larger genomic regions to achieve sufficient sampling. Genome signatures have been used to detect horizontally transferred DNA [36-39], reconstruct phylogenetic relationships [22,32,40] and infer lifestyles of bacteriophage [41,42].

Genome signatures also offer a compelling means of assigning metagenomic sequence fragments to microbial taxa, a procedure termed 'binning' [43]. This is a prerequisite for realizing some of the most valuable opportunities random shotgun metagenomics offers, including assignment of ecological and biogeochemical functions to particular community members and assessment of population-level genomic diversity and community structure. However, binning is a formidable challenge because: the inherent diversity of microbial communities typically limits genomic assembly, resulting in highly fragmentary data [13]; there are few universally conserved phylogenetically informative markers, leaving the vast majority of metagenomic sequence fragments 'anonymous' with regard to their organism of origin; and current sequence databases grossly under-represent the microbial diversity in the natural world, limiting the utility of fragment recruitment or BLAST-based methods [13,44,45]. Consequently, it is important to develop methods that classify all genome sequence fragments independently of reference databases.

Genome signatures are a promising approach for sequence classification. However, it is important to understand the source of the signal and how environmental effects and evolutionary distance will compromise it. To date, sequence signatures have been explored using genomes from cultivated microbes [22,30-34], and prospects for binning have been evaluated based largely on simulated datasets consisting of mixtures of isolate genomes [44,46-48]. Although these studies are indispensable in that they allow theoretical evaluation of binning capability, they do not represent the diversity (community-wide and within population) and dynamics (for example, horizontal gene transfer, recombination, viruses) of real microbial communities. Further, they employ genomes derived from disparate environments and so do not address the extent to which environmental factors shape genome signatures. It has been reported that environment shapes nucleotide composition [26,49-51]. If so, then genome signatures may not discriminate coexisting, coevolving organisms, especially where environmental pressures are extreme. On the other hand, binning results of real microbial communities [46,48,52] are inherently difficult to evaluate because the true identity of most sequence fragments is unknown. Thus, there remain fundamental questions regarding the forces and processes that give rise to and maintain genome signatures, and the extent to which these signatures are obscured by shared environmental pressures and community interactions such as horizontal gene transfer and broad host range viruses.

Here we present a comprehensive analysis of genome signatures in sequences derived from natural biofilms inhabiting a subsurface chemolithoautotrophic acid mine drainage (AMD) ecosystem in the Richmond Mine at Iron Mountain, CA [53]. The biofilms are dominated by just a handful of organisms that are sustained primarily by the oxidation of Fe(II) derived from pyrite (FeS_2_) dissolution [54]. Due to this relatively low diversity, modest levels of shotgun sequencing (approximately 100 Mb per sample) have yielded deep genomic sampling (10 to 20× sequence coverage) of the dominant populations, enabling reconstruction of 12 near-complete genomes from three samples [16,55,56] (BJ Baker *et al*., submitted). These assembled composite genomes provide the organism affiliation of sequences with which binning accuracy can be evaluated. Therefore, the dataset allows assessment of binning performance while capturing sequence heterogeneity that is an intrinsic feature of natural microbial populations. We find that AMD biofilm microorganisms are indeed distinguished by population-specific genome signatures and show that sequence signatures can be used to identify and cluster sequences from low-abundance community members *de novo*, without reference genomes or reliance on databases. Our results have implications for metagenomic binning and provide new insights into the sources of genome signatures that distinguish coexisting populations.

## Results

### Description of samples, community genomic sequencing and assembly

**Figure 1:**
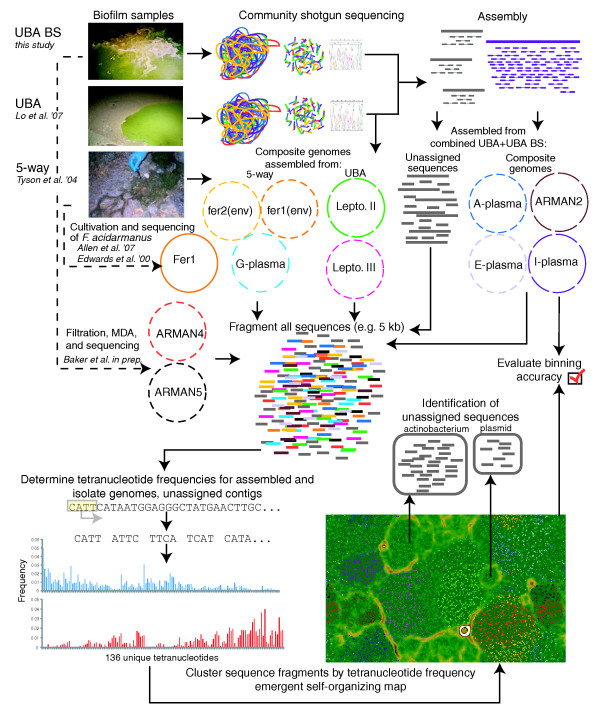
Overview of samples, data, and methods. MDA, Multiple Displacement Amplification. Lo *et al*. 2007 [55]; Tyson *et al*. 2004 [16]; Allen *et al*. 2007 [8]; Edwards *et al*. 2000 [57].

An overview of our methodology is shown in Figure 1. Community genomic sequence was obtained from two previously described biofilm samples from the UBA location of the Richmond Mine at Iron Mountain: a pink subaerial biofilm collected in June 2005 ('UBA') [55] and a thicker floating biofilm collected in November 2005 ('UBA BS') [12]. These two biofilms contained overlapping subsets of organisms in different proportions. The UBA biofilm was dominated by bacterial *Leptospirillum *spp. group II and group III (*Nitrospirae*) populations, for which near-complete genomes have been reconstructed [55,56]. The most abundant microorganisms represented in the UBA BS genomic data were from archaeal populations, including an uncultivated representative of a novel euryarchaeal lineage, ARMAN-2 [20], and A-plasma, E-plasma, and I-plasma, members of the order Thermoplasmatales. To facilitate reconstruction of genomes from these and other lower-abundance organisms, a combined assembly included unassigned sequences from UBA and all sequences from UBA BS. Random shotgun sequences derived from both ends of approximately 3-kb DNA fragments, and each fragment was likely sampled from a different individual cell with a potentially distinct genome sequence. Therefore, genome reconstructions represent composite sequences. However, single nucleotide polymorphism density was typically very low (< 0.3%). For a small subset of the many cases where there were subpopulations with different gene content, alternative genome paths were also reconstructed [9,55].

**Figure 2:**
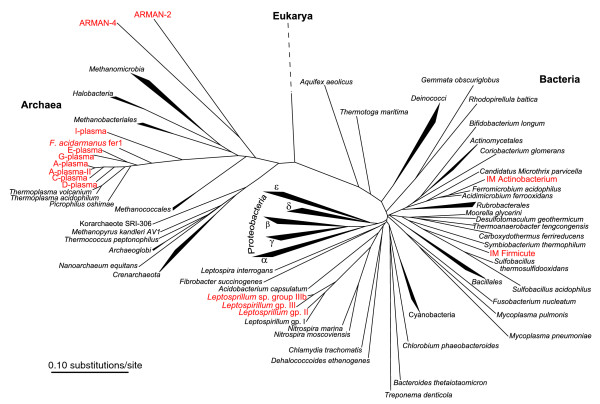
Phylogenetic tree of 16S rRNA gene sequences from Iron Mountain community genome sequencing (red) and selected sequences from cultivated organisms. *Ferroplasma *types I/II are not shown due to their near-identical sequences to *F. acidarmanus*. Sequences for which only partial coverage of the 16S rRNA gene was obtained are not shown, including ARMAN-5, a gammaproteobacterium, additional Actinobacteria, and *Sulfobacillus*-like sequences.

From the combined dataset, near-complete genomes were reconstructed for ARMAN-2, I-plasma, E-plasma, G-plasma, and A-plasma (Table 1). In addition to sequences that were assigned to these deeply sampled genomes, 14,700 sequences remained unassigned to any organism, including 7,030 contigs longer than 1.4 kb and 3,631 contigs longer than 2.0 kb. A number of shallowly sampled 16S rRNA gene-containing sequence fragments were recovered, indicating substantial sampling of diverse lower-abundance community members (Figure 2).

**Table 1:** Deeply sampled composite genomes from Iron Mountain community genomic datasets used in binning analysis

**Composite genome**	**Sample(s)**	**Sequence (Mb)**	Coverage*	**G+C content**	**Reference**
I-plasma^†^	UBA, UBA BS	1.69	20×	44	This study
E-plasma	UBA, UBA BS	1.58	9×	38	This study
A-plasma	UBA, UBA BS, UBA filtrate	1.94	8×	46	This study
G-plasma	5-way, UBA	1.78	8×	38	This study
*Leptospirillum *group II^†^	UBA	2.64	25×	55	[55]
*Leptospirillum *group II^‡^	5-way	2.72	20×	55	[9]
*Leptospirillum *group III^†^	UBA	2.82	10×	58	[56]
*Ferroplasma acidarmanus *fer1^†^	5-way	1.94	NA	37	[8]
*Ferroplasma *fer1(env)	5-way	1.46	4.5×	36	[8]
*Ferroplasma *fer2(env)	5-way	1.82	10×	37	[10]
ARMAN-2^†^	UBA, UBA BS	1.0	15×	47	Baker *et al*., submitted
ARMAN-4	UBA filtrate	0.81	8×	35	Baker *et al*., submitted
ARMAN-5	UBA filtrate	0.90	8×	35	Baker *et al*., submitted
Viral genomes	UBA, UBA BS	Variable	Variable	Variable	[12]

*Estimated sequence coverage (read depth). ^†^Genomes used for evaluation of binning performance on variable length fragments. ^‡^The *Leptospirillum *group II 5-way genome was included in some ESOM binning and was indistinguishable from the *Leptospirillum *group II UBA genome, but is not shown in Figure 2. NA, not applicable.

### Clustering sequences by tetranucleotide frequency and emergent self-organizing map

**Figure 3:**
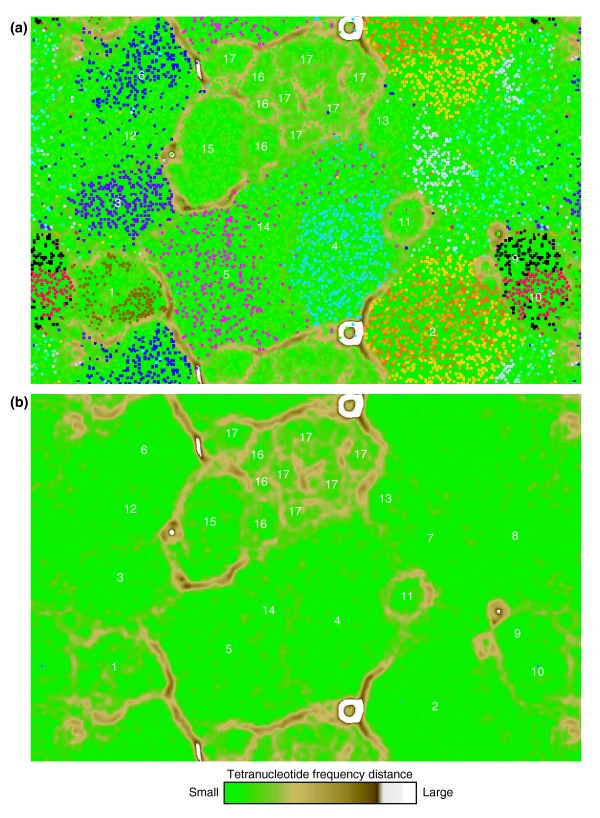
ESOM of genomic sequence fragments based on tetranucleotide frequency (5-kb window size; all contigs > 2 kb were considered). Note that the map is continuous from top to bottom and side to side. **(a) **Each point represents a sequence fragment; sequences whose origin is known (from assembly information) are colored as indicated below. Unassigned sequences are shown in green. Regions are numbered as follows: (1) ARMAN-2, brown; (2) *Ferroplasma *(*F. acidarmanus *fer1, dark orange; fer1(env), orange; fer2(env), light orange); (3) I-plasma, purple; (4) *Leptospirillum *group II, light blue; (5) *Leptospirillum *group III, pink; (6) A-plasma, navy blue; (7) E-plasma, light purple; (8) G-plasma, turquoise; (9) ARMAN-4, black; (10) ARMAN-5, red. Regions 11 to 17 are novel genomic regions identified in this study: (11) putative *Leptospirillum *plasmid; (12) A-plasma variant and C-plasma; (13) D-plasma; (14) *Leptospirillum *group III variant; (15) an actinobacterium; (16) mixed Actinobacteria; (17) mixed low-abundance bacteria, including *Sulfobacillu*s spp., other *Firmicutes*, and a gammaproteobacterium. **(b) **Topography (U-Matrix) representing the structure of the underlying tetranucleotide frequency data from (a). 'Elevation' represents the difference in tetranucleotide frequency profile between nodes of the ESOM matrix (see legend); high 'elevations' (brown, white) indicate large differences in tetranucleotide frequency and thus represent natural divisions between taxonomic groups.

We constructed a dataset that contained all sequences from the combined assembly (assigned and unassigned), previously assembled composite genome sequences, and the genome sequence from *Ferroplasma acidarmanus *fer1, which was cultivated from AMD solutions in the Richmond Mine [8,57] (Figure 1, Table 1). To analyze the distribution of genome signatures among and between populations, all contigs and assembled genomes were fragmented into 5-kb pieces, then pooled and clustered by self-organizing map (SOM) [58] based on tetranucleotide frequency distributions (Figure 1; see Materials and methods for details). The SOM is an unsupervised neural network algorithm that clusters multidimensional data and represents it on a two-dimensional map. SOMs of tetranucleotide frequencies have been used previously to successfully bin sequence fragments from isolate genomes [33,59] and some environmental samples [46,48,52]. We utilized an implementation of the SOM, emergent SOM (ESOM), which is distinguished by its use of large borderless maps (for example, thousands of neurons) and visualization of underlying distance structure with background topography [60]. This visualization, where map 'elevation' represents the distance in tetranucleotide frequency between data points, is referred to as the U-Matrix [60]. Thus, genomic clusters were visualized not only by the cohesive clustering of fragments from each genome, but also by distance structure whereby barriers between clusters represent the large differences in genome signatures between genomes relative to those within genomes (Figure 3). This visualization of genomic clustering was used to evaluate the accuracy of the binning based on assembled genomes and to identify novel regions of sequence signature space.

**Figure 4:**
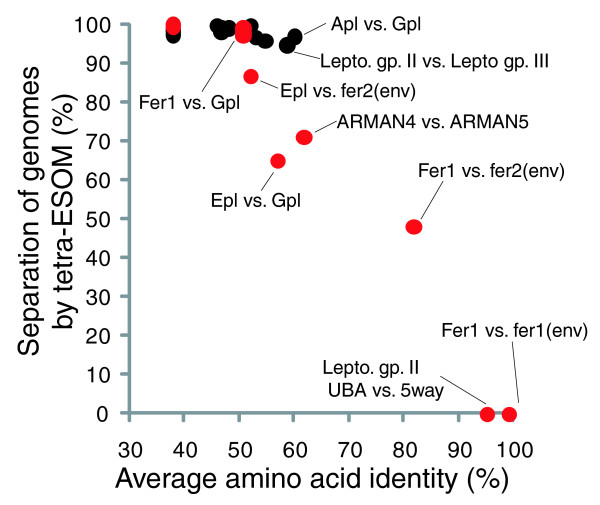
Ability of tetra-ESOM to resolve AMD populations as a function of evolutionary distance (average amino acid identity) and %GC. Black points represent comparisons between genomes with different %GC (> 2% different), red points are genome pairs with < 2% different %GC. These data were collected using a 5-kb window size and 2-kb cutoff length.

Inspection of the clustering results in light of assembly information provided a broad measure of the ability of tetranucleotide frequency-based ESOM (tetra-ESOM) to resolve sequences from coexisting populations of the community. To quantify the degree of segregation of fragments from genomes at various evolutionary distances, we adapted a method using fixed point kernel densities (Figure 4; Additional data file 1). We found that sequence fragments from closely related strains or species could not be distinguished. For example, two strains of *F. acidarmanus *sharing 97% average nucleotide identity (fer1 and fer1(env) [8]) mapped directly on top of each other, as did two types of *Leptospirillum *group II, which share 95% average nucleotide identity [55] (only one type of Leptospirillum group II is shown in Figure 3 for this reason; Figures 3 and 4). Sequences from *Ferroplasma *types I and II, which share 83% average nucleotide identity and are known to participate in homologous recombination [10], were segregated to some extent by tetra-ESOM, but type II was split and there was no well-defined boundary between the two types. Good separation of *Leptospirillum *groups II and III was achieved, except for certain genomic regions containing mobile elements, as described further below. Among members of the Thermoplasmatales, populations were distinguished by genome signatures but borders were variably well-defined (Figure 3). In particular, G- and E-plasma were not well resolved. I-plasma, which is quite divergent from the other Thermoplasmatales (Figure 2), was the only member of the Thermoplasmatales for which a distance-based border was clearly delineated. Although genomes with similar %GC were generally more difficult to separate, several genomes with near-identical %GC were easily separated (for example, G-plasma versus *Ferroplasma*) (Figures 3 and 4).

To quantitatively evaluate binning performance on sequence fragments of different lengths, tetra-SOMs were run on the same dataset (including unassigned sequences and reconstructed composite genomes) but with sequences broken into various fragment sizes. Binning accuracy was calculated for a subset of genomes for which deeply sampled and manually curated assemblies are available (Additional data file 2). For sequence fragments 5 kb or larger, sensitivity (percentage of fragments from each genome correctly identified) and precision (percentage of fragments in each bin belonging to the correct genome) rates of > 90% were achieved (Additional data file 2). Sensitivity was somewhat lower for *Leptospirillum *groups II and III due to poor resolution of certain genomic regions between these two populations. When *Leptospirillum *was considered as a single group, binning sensitivity was comparable to the other reference genomes. Sensitivity decreased notably only when shorter (< 5 kb) sequence fragments were analyzed, but precision remained remarkably high even for 1,400-bp fragments (Additional data file 2). Lower sensitivity is due to sequence fragments that fall between clusters, beyond the borders of any bin. Notably, the tetra-ESOM correctly assigned sequence fragments as short as 500 bp, provided that some larger fragments were included in the analysis (Additional data file 2b). To address the question of how genome completeness influences performance, genomes randomly subsampled at different levels were analyzed by tetra-ESOM. Binning accuracy was maintained even at 20% genome sequence; only at 10% subsampling was a notable decline observed, and even then only for certain genomes (Additional data file 3).

Incorrectly assigned fragments often contained mobile elements or other features expected to have atypical nucleotide composition. The majority (54 of 94) of incorrectly binned fragments from all five reference genomes show evidence of transposons, prophage, or integrated plasmids. Other frequently unresolved genomic regions contain CRISPR elements [61] and rRNA genes, both of which have constrained sequences and thus atypical tetranucleotide patterns [62]. The region of the ESOM map containing a mixture of *Leptospirillum *groups II and III (Figure 3) was dominated by fragments (80 of 92) encoding mobile elements that may be exchangeable between the two *Leptospirillum *groups (for example, integrated plasmid-like sequence [56]) and strain/group-unique regions believed to have been recently acquired (for example, prophage).

Interestingly, many strain-unique regions were correctly binned with their host genomes. There are 197 strain-unique genes between the fer1 and fer1(env) genomes, the majority of which occur in distinct genomic blocks of up to 24 genes with atypical %GC content inferred to be the result of prophage insertion [8]. Ninety-six percent (22 of 23) of sequence fragments containing these genomic islands were accurately assigned as *Ferroplasma *in our binning analysis.

### Genome signatures of low-abundance community members and viruses

The tetra-ESOM revealed large regions of the map that were devoid of sequence fragments of known organism affiliation (Figure 3, regions 11 to 17). We used mate pair linkage with rRNA gene-containing contigs, phylogenetic analysis, and/or close relatedness (synteny and identity) to other community members to identify these bins as follows: a new type of *Leptospirillum *most closely related to *Leptospirillum ferrodiazotrophum *(group III); several members of the Thermoplasmatales for which genomic sequence had not been previously obtained (C-plasma, D-plasma, and a divergent type of A-plasma); several Actinobacteria; and multiple more shallowly sampled populations, including a gammaproteobacterium and several *Sulfobacillus*-like organisms (Figures 2 and 3). A small, prominent region of the map adjacent to the *Leptospirillum *groups contained approximately 250 kb of composite sequence (Figure 3, region 11) inferred to be a *Leptospirillum *plasmid [56]. Tetranucleotide usage patterns of this putative plasmid are quite distinct from those of either *Leptospirillum *groups (Additional data file 4).

We calculated tetranucleotide frequencies for viral genomes that were recently reconstructed from the same genomic datasets and linked to their hosts via CRISPR viral resistance system sequences (Additional data file 4) [12]. Three of the viruses closely resemble their hosts' tetranucleotide usage (AMDV1, *Leptospirillum *groups II and III; AMDV4, E-plasma; AMDV3, A-/E-/G-plasma), a trend that has been observed previously for cultivated viruses and hosts [41,63]. Interestingly, two viruses have very different tetranucleotide frequency patterns (AMDV2, E-plasma; AMDV5, I-plasma; Additional data file 4).

### Characteristics of genome signatures

**Figure 5:**
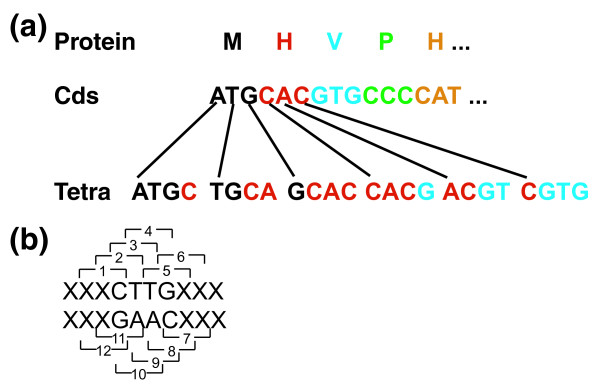
Schematic of how tetranucleotide frequency relates to reading frame and potential codons. **(a) **Tetranucleotide frequencies are calculated independently of reading frame with a 1-bp sliding window; thus, they may sample a complete codon or span two partial codons. **(b) **Because reverse complementary pairs are summed together, both strands are sampled. Therefore, depending on the coding strand and reading frame, there are 12 potential codons sampled by each tetranucleotide.

As expected, the frequency at which each tetranucleotide occurs is related to overall %GC: GC-rich tetranucleotides are abundant in high-GC genomes and uncommon in low-GC genomes. However, patterns of tetranucleotide usage extend beyond trends in %GC (Additional data file 4) and genomes with near-identical %GC were effectively segregated by tetra-SOM. Because tetranucleotide frequencies are calculated with a 1-bp sliding window and reverse complementary pairs of tetranucleotides are summed together, all possible reading frames on both strands are sampled. In addition to spanning complete single codons, adjacent pairs of partial codons are also sampled (Figure 5). Therefore, tetranucleotide frequency captures amino acid composition and synonymous codon usage, as well as information regarding avoidance of certain adjacent codons ('codon pair bias' [64]).

To assess the contributions of these potential sources of genome signature signal, we compared SOMs based on amino acid composition, codon composition, and tetranucleotide frequency. Amino acid composition alone distinguished certain genomes (Additional data file 5). This was especially true for phylogenetically distant organisms (for example, archaea versus bacteria), but some separation was also apparent among groups within some lineages such as *Ferroplasma *versus other Thermoplasmatales. SOMs based on codon composition were notably more accurate than amino acid composition and comparable to those based on tetranucleotide frequency (Additional data file 5).

**Figure 6:**
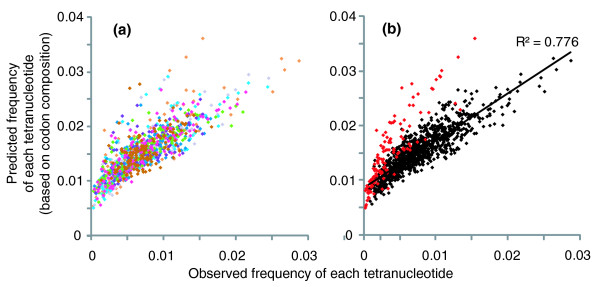
Tetranucleotide frequency predicted by codon abundance (a weighted average of the frequencies of the 12 potential codons associated with each tetranucleotide) versus observed tetranucleotide frequency. **(a) **Color indicates the genome of origin (using the same color scheme as Figure 3). **(b) **Palindromic nucleotides are indicated in red. R^2 ^indicates the square of the Pearson correlation coefficient.

Additional features of the relationship between codon composition and tetranucleotide frequency were revealed by comparing the observed frequency of tetranucleotides to the frequency predicted from genome-wide codon usage (see Materials and methods). Observed and predicted tetranucleotide frequency correlated strongly (Figure 6), and differences in the frequencies of individual tetranucleotides between genomes are correlated with differences in corresponding codon usage between genomes (Additional data file 6). Exceptions to this trend are primarily palindromic tetranucleotides that occur less frequently than predicted (Figure 6b). Five of the 16 possible palindromic tetranucleotides are most strongly and consistently underrepresented: AATT, ATAT, TATA, GATC, and GGCC. The extent to which palindromic tetranucleotides are avoided in both viral and microbial genomes varies significantly and thus could be a factor in defining genome signatures (Additional data file 4). To test this possibility, we visualized the SOM distance structure for only one tetranucleotide at a time and found that certain palindromic tetranucleotides (GATC, TATA, ATAT) are particularly informative in distinguishing members of the Thermoplasmatales that share near-identical %GC (*Ferroplasma *types I and II, G-plasma, E-plasma). However, SOMs run excluding all 16 palindromic tetranucleotides distinguished populations with accuracy comparable to that achieved using all tetranucleotides, indicating that palindrome avoidance is not a primary component of the genome signature.

The correlation of genome signatures with codon usage raises the question of whether they persist in intergenic regions. Thus, we extracted intergenic regions from assembled and annotated genomes and analyzed them with coding regions by tetra-ESOM (intergenic regions were concatenated to tally tetranucleotide frequencies but care was taken to avoid artifacts; see Materials and methods). Intergenic regions from each genome formed discrete, cohesive clusters that mapped adjacent to coding regions from the same genome but were separated by U-Matrix boundaries (Additional data file 7). Intergenic sequences from each genome were grouped based on length, concatenated, and analyzed by ESOM; all size classes of intergenic regions from the same genome clustered together regardless of length, from the shortest (4 to 20 bp) to longest (> 1,000 bp) (data not shown). The noncoding complement of each Thermoplasmatales genome formed a distinct cluster adjacent to noncoding regions of the other Thermoplasmatales. The only outlier to this trend was A-plasma, which has the highest %GC among these organisms. Based on U-Matrix background, the distance between noncoding sequences of different genomes is comparable to the distance between noncoding and coding sequences of the same genome. To determine if the presence of noncoding sequence influences binning accuracy in the initial experiments, we calculated the percentage of coding sequence on incorrectly binned fragments from the five reference genomes (5 kb and 1 kb window sizes). For many genomes, the incorrectly binned fragments do indeed have a smaller average percentage of coding sequence. However, this percentage varied widely on incorrectly binned fragments. Only a small fraction of such fragments had a percentage of coding sequence smaller than one standard deviation below the genome-wide average (Additional data file 8).

For sequence signatures to differentiate populations in a genome-wide manner, it is necessary that within-genome differences resulting from atypical regions of amino acid and/or synonymous codon usage are smaller than between-genome differences. This issue is especially relevant in AMD, where proteins are under diverse constraints depending on whether they function in the extracellular (around pH 1) or intracellular (around pH 5) environment [65]. Indeed, proteins from the AMD populations in these two fractions have disparate isoelectric points owing to the unique amino acid composition of acid-stable proteins [66]. We identified 106 *Leptospirillum *group II-UBA proteins that are consistently enriched in the extracellular fraction according to environmental shotgun proteomics data [55,66] and compared sequence signatures of their genes with the other 2,522 *Leptospirillum *group II genes. No systematic differences were detected via tetra-ESOM, suggesting that genome signatures persist even when gene sequences are influenced by considerable protein-coding constraints (Additional data file 9).

Selection for codons that optimize translation rate may also influence codon usage. We analyzed genome signatures for the 50 *Leptospirillum *group II proteins most abundantly detected via environmental shotgun proteomics [55,66]. With the exception of one subset of genes encoding mainly ribosomal proteins (which mapped into the mixed region between *Leptospirillum *groups II and III), highly expressed genes clustered with the rest of the genome (Additional data file 9).

## Discussion

Through analysis of a deeply sampled and extensively curated community genomic dataset, we have demonstrated that genome signatures can be used to differentiate coexisting microbial populations despite functional and environmental constraints, processes such as lateral gene transfer, and pressures imposed by viral predation that might have diminished them to the point that they are no longer diagnostic. The genome-wide nature of the signatures makes them potentially useful for classification of sequence fragments. Results from our AMD dataset show that the signal can be detected on fragments as small as 500 bp, genome clusters can be defined using fragments as short as 1,400 bp (Additional data file 2) and a small fraction of the genome (Additional data file 3). These findings suggest broad applicability of the tetra-ESOM approach for metagenomic studies. However, in order to understand and predict its utility for binning, it is important to identify sources of genome signatures as well as processes that are likely to diminish the signal.

### Insights into the sources of distinctive genome signatures

It has been suggested that environmental constraints strongly shape nucleotide composition [26,49-51]. If this were the case, two effects should be apparent in genome signatures of AMD populations. First, shared pressures deriving from the extreme AMD environment would drive genome signatures together, potentially obscuring differences between populations. Second, since each genome encodes proteins destined for diverse environments (that is, intracellular and extracellular), there should be prominent intra-genome variation of genome signature and scattering of fragments from the same genome into disparate regions of the SOM. Neither of these expectations is met in the AMD dataset. There are vast differences in nucleotide composition between populations, with genomic %GC ranging from 35% (ARMAN-4 and ARMAN-5) to 69% (low-abundance Actinobacteria) and genome signatures forming discrete clusters. Amino acid compositional constraints required for stability of proteins exposed to acidic solutions do not result in sequence signatures that are markedly distinct from the rest of the genome. In other words, within-population differences in genome signature are small relative to differences between populations. Although we do not rule out some environmental influence on genome signatures, we conclude that, in AMD, this influence is not strong enough to obscure differences between populations. Similar community-wide analyses need to be conducted in other systems to determine whether our findings extend to other extremophilic microbial communities.

Our results show that genome signatures are related to several traits, including %GC, amino acid composition, synonymous codon usage, and palindrome avoidance. These characteristics are interrelated and further connected to a host of biochemical, ecological, and evolutionary processes (Additional data file 10). Large differences in %GC and/or amino acid composition guarantee distinctive genome signatures but are not required to differentiate genomes. At finer evolutionary scales, where %GC and amino acid composition are not informative, populations can be readily distinguished through subtle differences in tetranucleotide frequency, which correlate with genome-specific synonymous codon usage. Tetra-ESOM analyses based on codon usage and tetranucleotide frequency displayed similar clustering resolution, indicating that little signal derives from longer-range characteristics such as codon pair bias. It should be noted, however, that using tetranucleotide frequency rather than codon composition has practical advantages for binning because it is independent of coding strand and reading frame and thus insensitive to errors in gene-calling or frame shifts due to poor quality sequence. These issues are particularly important for short, low-coverage sequence fragments.

Although genome signatures are largely manifested through codon composition, the observation that population-specific signatures also occur in non-coding regions (Additional data file 7) suggests a mechanism of generation that is independent of protein coding. We hypothesize this underlying process is mutational bias associated with DNA replication and repair, which exerts directional pressure on nucleotide composition [24]. In fact, between-genome codon biases can be predicted solely by %GC and context-dependent nucleotide biases (that is, mutation rates at each site are dependent on the identity of neighboring nucleotides) calculated from non-coding regions [67,68]. It is interesting to note that non-coding regions mapped into discrete clusters, distinct from coding regions of the same genome or non-coding regions of different genomes, including those with identical %GC. Differences in genome signature of coding and non-coding sequences from the same genome are to be expected based on differing functional constraints on these regions (for example, coding amino acids versus small RNAs or regulatory elements such as promoters). The distinction of non-coding regions from different genomes is consistent with genome-specific mutational biases.

An alternative to the mutation bias hypothesis, at least for coding sequences, is that genome signatures are shaped by factors related to translation. Changes in codon usage can be driven by changes in the tRNA gene complement [69,70] that may occur, for example, through interaction with plasmids and viruses [71]. However, we found AMD genomes with distinct genome signatures, such as G-plasma, E-plasma, and *Ferroplasma*, that have only minor differences in tRNA gene content, and these differences do not correspond to observed differences in codon usage. In addition to tRNA gene complement, there may be changes in tRNA gene regulation, which can significantly impact cellular tRNA concentrations and have been correlated with changes in codon usage [72]. Thus, although we cannot rule out a tRNA regulatory influence on genome signatures, our findings suggest that coevolution of tRNA gene content and codon usage is not a primary mechanism underlying the divergence of genome signatures in related AMD populations.

Codon bias can also arise as the result of selection for certain codons that are optimal for fast and/or accurate translation [73]. This form of codon bias primarily influences the subset of genes encoding highly expressed proteins, is prevalent for fast-growing organisms [69,74], and correlates with ecological strategy [75]. In fact, a *Leptospirillum *group II genome fragment encoding nine ribosomal proteins and two translation elongation factors had distinctive tetranucleotide composition, indicating that this mode of codon bias occurs in AMD organisms. However, as commonly construed, translational selection would influence within-genome codon bias, not the genome-wide codon biases that differentiate populations as observed in our study. It is tempting to speculate that differences in ecological strategy (for example, response rate to resource availability [76]) could have genome-wide influence on codon usage, but there is currently no evidence in our dataset to suggest that this is the case.

Finally, restriction avoidance places another selective genome-wide constraint on DNA composition that may contribute to genome signatures. Under-representation of palindromic tetranucleotides (Figure 6) has been attributed to avoidance of enzymes designed to recognize and degrade foreign DNA [22,32,46]. Our data show that palindrome avoidance contributes to the genome signature but is not the sole or even primary determinant. Most archaeal viruses and bacteriophage have sequence signatures that resemble their hosts, including avoidance of specific subsets of palindromes. However, mismatches between the tetranucleotide signatures of AMDV2 and AMDV5 and their respective hosts point to the lesser importance of palindrome avoidance in these organisms. In the case of AMDV5, other evidence suggests a recent alteration in host range [12]. It is interesting to note that the genomes of archaeal AMD viruses encode several restriction modification (RM) system genes. These may have significance for virus host-interactions [77] and for influencing genome signatures. Broad host range viruses or viruses that jump to new hosts can potentially drive changes in the host sequence signatures if they replace or supplement the restriction systems of the host. Alternatively, the degree of similarity in tetranucleotide signatures of viruses and their hosts may be a function of the extent to which the virus relies upon its host's replication and translation machinery (for example, associated with a lysogenic versus lytic lifestyle) [41,42,63].

### Implications for metagenomic, ecological, and evolutionary studies

Due to the high levels of diversity in most natural systems, random sequencing approaches yield fragmentary data, often comprising genomic sequences no more than a few kilobases in length. While more comprehensive coverage of individual organisms can be achieved by single cell genomics [78-80] or targeted, large-insert approaches [81,82], random shotgun approaches retain two important advantages: the random nature provides insights that are unbiased by preconceived notions of community composition; and population-level variation is captured because each sequencing read derives from a different individual cell.

A key challenge for virtually all shotgun metagenomics investigations is the assignment of genome fragments to the organism they derive from. This step links organism to metabolism and function and is essential if we are to understand microbial community dynamics and predict ecosystem level impacts of changes in community membership and structure. Binning is particularly challenging for lower-abundance organisms, which may play keystone roles that are critical to ecosystem function. Thus, our finding that tetra-ESOM can resolve the phylogenetic affiliation of genome fragments on the scale of two mate-paired reads is of great significance. This approach has clear applicability to low-complexity datasets such as those derived from our AMD biofilms, bioreactors [83], and enrichment cultures [84]. In fact, even for the relatively extensively analyzed AMD dataset, it revealed multiple new genomic clusters, including a near complete genome of a novel actinobacterium (GJ Dick *et al*., in preparation), a putative plasmid, and many discrete but less well-sampled populations.

Tetra-ESOM may also provide a powerful method for analysis of unassembled data from complex samples such as soil, seawater, and the human microbiome if representative isolate genomes are available. The feasibility of binning metagenomic sequences from complex samples using reference genomes will increase with current initiatives to fill in the phylogenetic tree with genome sequences from cultivated microorganisms.

An important advantage of unsupervised, compositional-based approaches such as tetra-ESOM is that gene sequences need not be represented in databases to be identified; only representation of the genome signature is required. This is in contrast to fragment recruitment [13] and BLAST-based binning approaches that only work for homologous sequences. We found that clusters of a few hundred kilobases of sequence (as little as 20% of the genome) were resolved, suggesting that a few fosmids or bacterial artificial chromosomes linked to 16S rRNA genes can be sufficient to serve as a reference to define a bin. Thus, recent progress in using large-insert metagenomic libraries to link 16S rRNA genes to genomic sequence from diverse uncultivated microorganisms is very valuable in this regard [85].

Because the reach of composition-based approaches to binning extends beyond gene content of reference genomes, they hold great promise for identifying and classifying genes from the variable fraction of the pan-genome (present in only a subset of strains or species), an important determinant of pathogenicity and niche differentiation [86-88]. In AMD populations, genome reconstruction has shown that this strain-variable fraction often involves inserted plasmid and virus sequences [8,9]. In the current study, these integrated elements clustered either with the host genome or in regions shared between different species or genera. Since horizontally transferred DNA is rapidly converted to the genome signature of its new host [22,28,89], the extent to which such genomic regions reflect the genome-wide signature of nucleotide composition is likely a function of the donor of the genetic material and how recently they were acquired. Recently acquired sequences with distinctive tetranucleotide patterns may bin incorrectly, and unexpected binning outcomes can be used to identify laterally transferred regions [62,90].

Although the tetra-ESOM method works well to separate sequence fragments from organisms distinct at the genus or higher level, it has some limitations. Tetra-ESOM is generally unable to distinguish closely related species or strains. An important question, especially for more diverse samples, is whether limitations in genome sequence signature space will impose an inherent constraint on the number of populations that can be resolved. There are a staggering 6 × 10^222 ^ways to code for a typical protein in our samples (based on an average protein size of 467 amino acids and assuming an average of 3 possible ways to code for any amino acid). This richness of protein coding space suggests ample capacity for numerous genome signatures. To date, SOMs have shown promising results in resolving up to 81 complete genomes, in successfully classifying fragments of 1,502 genomes into phylogenetic groups, and in visualizing phylogenetic clustering of sequences in complex environmental samples [46]. However, it remains difficult to assess the accuracy and phylogenetic resolution of oligonucleotide-based SOMs on metagenomic datasets from diverse natural microbial communities. Another concern is computational demand. Continued increases in processor speeds will likely need to be supplemented with more efficient and/or accurate algorithms such as the recently introduced hyperbolic SOM [91] and growing SOM [59].

## Conclusions

Bacterial, archaeal, and viral populations in the AMD biofilm community have genome-wide signatures of nucleotide composition that are effectively captured and visualized through self-organizing maps of tetranucleotide frequency. We conclude that even under extremely acidic conditions, shared environmental pressure does not obscure genome signatures of nucleotide composition. Our data point to pervasive mechanisms of generating and maintaining genome signatures; although a variety of factors and processes contribute, we propose that mutational bias is the primary underlying mechanism driving the divergence of genome signature between closely related organisms. The resulting signal, evident through synonymous codon usage, is genome-wide and sufficiently diagnostic to classify fragmentary metagenomic data from coexisting populations of a natural microbial community at approximately the genus level. However, distinguishing features of genome signatures may be subtle, being masked by within-genome heterogeneity and the multidimensional nature of tetranucleotide frequency patterns. Tetra-ESOM is a key method for visualizing and exposing these potentially weak signals. Being unsupervised, it requires no database representation of the organisms present. Visualization of the data structure highlights differences between populations and reveals atypical regions corresponding to biologically meaningful genomic features such as mobile elements or previously unrecognized genotypes present at low abundance in the community. When employed in conjunction with complementary methods such as genomic assembly and analysis of phylogenetic marker genes, genome signatures offer powerful perspectives on metagenomic data.

## Materials and methods

### Sample collection, construction of genomic libraries, sequencing, and community genomic assembly

An overview of the samples and methodology used in this study is provided in Figure 1. Sample collection, DNA extraction, random fragmentation and cloning of approximately 3-kb fragments, Sanger sequencing, assembly, and curation of community genomics data were performed using phred/phrap/consed package as detailed previously [12,55]. The combined UBAs nonLeptos dataset was constructed by assembling sequencing reads derived from both the UBA BS and UBA biofilm samples (with UBA reads previously assigned to *Leptospirillum *spp. removed). This included 229,082 reads and approximately 210 Mb of total sequence, which assembled into 15,929 contigs and 36.6 Mb of composite sequence.

### Phylogenetic analysis

The phylogenetic tree of 16S rRNA genes was constructed by neighbor joining (default parameters) with the ARB software package [92] and 'SILVA SSU ref' database [93].

### Calculation of tetranucleotide frequencies and clustering by ESOM

Tetranucleotide frequencies were determined for each assembled contig using a custom Perl script. Frequencies were calculated with a 1-bp sliding window and pairs of reverse complementary tetranucleotides were summed in order to avoid strand bias. Longer contigs and assembled genomes were split into 5-kb windows and only contigs longer than 2 kb were considered unless noted otherwise. To assess binning accuracy, data points (representing contigs/windows) are colored according to their genome of origin (when known), but this information is not available to the clustering process.

Contigs were clustered by tetranucleotide frequency utilizing Databionics ESOM Tools [94]. The input for tetra-ESOM was a 136-dimensional vector (representing the frequencies of the 136 unique reverse complement tetranucleotide pairs, normalized for contig length) for each contig/window. These raw frequencies were transformed with the 'Robust ZT' option built into Databionics ESOM Tools, which normalizes the data using robust estimates of mean and variance. Data were permuted before each run to avoid errors due to sampling order. Maps were toroidal (borderless) with Euclidean grid distance and dimensions scaled from the default map size (50 × 82) as a function of the number of data points, to a ratio of approximately 5.5 map nodes per data point. For example, a typical clustering with approximately 7,500 data points was run on map with dimensions 155 × 255. Training was conducted with the K-Batch algorithm (k = 0.15%) for 20 training epochs. The standard best match search method was used with local best match search radius of 8. Other training parameters were as follows: Gaussian weight initialization method; Euclidean data space function; starting value for training radius of 50 with linear cooling to 1; starting value for learning rate of 0.5 with linear cooling to 0.1; Gaussian kernel function.

### Clustering resolution versus evolutionary distance

To quantify the degree of clustering between closely related genomes, we analyzed SOM maps using fixed point kernel densities [95]. Spatial data from the SOM was imported into ArcGIS (ESRI Software) and clusters were defined using Hawth's Analysis Tools for ArcGIS [96]. Cluster boundaries were determined using density estimators that captured 90% of data points from each genome (Additional data file 1). We then calculated separation between genomes as a percentage (Non-overlapping points/Total number of points) for two bins being compared. Average amino acid identity was calculated as described previously [1].

### Predicted tetranucleotide frequency

The predicted frequency of each unique pair of reverse complementary tetranucleotides was calculated based on genome-wide frequencies of potentially contributing codons. As shown in Figure 5, for any given tetranucleotide there are 12 potentially associated codons depending on coding strand and reading frame. Four codons (numbers 3, 4, 9, and 10 in Figure 5) are fully captured by the tetranucleotide, four are partially captured at two of three positions (numbers 2, 5, 8, and 11), and four are partially captured at one of three positions (codons 1, 6, 7, and 12). Each of these three classes is weighted according to their contribution: 1, 2/3, and 1/3 respectively. For partially captured codons, contributions of all possibilities were taken into account; for example, in Figure 5, codon number 5 (TGX) there are four possible codons - TGA, TGT, TGC, and TGG.

### Binning performance on variable length sequence fragments and subsampled genomes

Sensitivity (percentage of fragments from each genome correctly identified) and precision (percentage of fragments in each bin belonging to the correct genome) of binning were calculated for a subset of assembled genomes that are deeply sampled and manually curated (Table 1; Additional data file 2). Fragment size was varied in two ways: all contigs were broken into a given size (2, 4, 6, or 10 kb); or 10% of each genome was randomly selected and fragmented (0.5, 1.0, 1.5, or 2.0 kb) while the remaining fraction of the genome was fragmented into 5-kb windows (Additional data file 2). Bin territories were defined manually, using boundaries apparent via distance-based background topology (U-Matrix) as guidelines. It is important to note this method allows data points between bins or near borders to remain unclassified. Analysis of subsampled genomes was conducted with assembled genomes only - unassigned fragments were excluded to prevent them from contributing to definition of bins. Genomes were fragmented into 5-kb sequences, which were then randomly selected to obtain the indicated percentage of the genome.

### Sequence signatures in coding versus non-coding regions

Intergenic regions were extracted and concatenated, with 'N's inserted between regions to avoid generation of erroneous tetranucleotides. Intergenic regions were grouped by size (in 20-bp bins) to monitor variance in sequence signatures from intergenic regions of differing lengths. All coding sequences were similarly concatenated with interleaving 'N's. Concatenated coding and non-coding regions were then broken into 5-kb windows and run against the same background dataset of assembled genomes and unassigned sequences as usual.

### Sequence signatures in extracellular and highly expressed protein-coding genes

Shotgun proteomics data were obtained for *Leptospirillum *group II extracellular and whole cell fractions from the ABend, ABfront, and UBA locations of the Richmond mine [55,66]. Proteins were defined as enriched in the extracellular fraction if, in at least two of the three samples, they were only detected in the extracellular fraction, or the ratio of spectral counts from extracellular to intracellular fraction was > 2. The 50 most abundantly expressed proteins were identified on the basis of tandem mass spectrometry (MS/MS) spectral counts. ESOM analysis of genes encoding extracellular and highly expressed proteins were both conducted as described above; open reading frames were concatenated, interleaved with 'N's, then split into 5-kb windows and analyzed along with the full dataset.

### Nucleotide sequence accession numbers

This Whole Genome Shotgun project has been deposited at DDBJ/EMBL/GenBank under the project accessions ACXJ00000000 (unassigned contigs), ACXK00000000 (A-plasma), ACXL00000000 (E-plasma), ACXM00000000, (I-plasma), and ACVJ00000000 (ARMAN-2, described in detail in BJ Baker et al., in preparation). The versions described in this paper are the first versions, ACXJ01000000, ACXK01000000, ACXL01000000, ACXM01000000, and ACVJ01000000.

## Abbreviations

AMD: acid mine drainage; ESOM: emergent self-organizing map; %GC: percentage content of guanine plus cytosine; SOM: self-organizing map.

## Authors' contributions

GJD, AFA, SLS, and JFB conceived and designed the experiments. GJD, BJB, SLS, APY, and BCT performed the experiments. GJD, AFA, SLS, BCT, BJB, APY, and JFB analyzed the data. GJD and JFB wrote the paper.

## Additional data files

The following additional data are available with the online version of this paper: a figure showing automated clustering of tetra-ESOM data using fixed point kernel densities (Additional data file 1); an evaluation of binning accuracy based on deeply sampled metagenomes for which contigs are assigned to genomes with a high degree of confidence (Additional data file 2); binning accuracy calculated for genomes that were sampled to varying extents of completeness (10 to 100%) (Additional data file 3); a heat map of average genome-wide frequency of each tetranucleotide for each genome, including bacteria, archaea, viruses, and a putative plasmid (Additional data file 4); comparison of tetra-ESOMs of assembled genomes based on amino acid composition, codon composition, and tetranucleotide frequency (Additional data file 5); a figure showing that the observed difference in frequency of each tetranucleotide between pairs of genomes correlates with the difference predicted based on codon composition (Additional data file 6); a figure showing tetra-ESOM of deeply sampled genomes for which coding and noncoding regions were separated (Additional data file 7); a figure showing for incorrectly binned fragments the percentage of sequence coding for genes in comparison with the genome-wide coding percentage (Additional data file 8); a figure showing tetra-ESOM of *Leptospirillum *group II genes coding for highly expressed proteins or proteins enriched in the extracellular fraction analyzed as separate fractions from the rest of the genome (Additional data file 9); a schematic of processes and factors influencing genome signature (Additional data file 10).

## Supplementary Material

Additional File 1 Automated clustering of tetra-ESOM data using fixed point kernel densities. Click here for file

Additional File 2 Evaluation of binning accuracy based on deeply sampled metagenomes for which contigs are assigned to genomes with a high degree of confidence. Click here for file

Additional File 3 Binning accuracy calculated for genomes that were sampled to varying extents of completeness (10 to 100%). Click here for file

Additional File 4 Heat map of average genome-wide frequency of each tetranucleotide for each genome, including bacteria, archaea, viruses, and a putative plasmid. Click here for file

Additional File 5 Comparison of tetra-ESOMs of assembled genomes based on (a) amino acid composition, (b) codon composition, and (c) tetranucleotide frequency Click here for file

Additional File 6 The observed difference in frequency of each tetranucleotide between pairs of genomes correlates with the difference predicted based on codon composition. Click here for file

Additional File 7 Tetra-ESOM of deeply sampled genomes for which coding and noncoding regions were separated. Click here for file

Additional File 8 Percentage of sequence coding for genes in comparison with the genome-wide coding percentage for incorrectly binned fragments. Click here for file

Additional File 9 Tetra-ESOM of *Leptospirillum *group II genes coding for highly expressed proteins or proteins enriched in the extracellular fraction analyzed as separate fractions from the rest of the genome. Click here for file

Additional File 10 Processes and factors influencing genome signature. Click here for file
