# Supplementary material for: Community-wide analysis of microbial genome sequence signatures
Source: Genome Biol. 2009 Aug 21;10(8):R85. doi: 10.1186/gb-2009-10-8-r85 (PMC2745766; doi:10.1186/gb-2009-10-8-r85)

Additional data file 1. Evaluation of genome sequence fragment clustering based on fixed point kernel densities. Shown is the ESOM map presented in Fig. 3, without the U-matrix background displayed. Data points of known identity (from assembly information) are enlarged and colored; others are unassigned. Contour lines are delineated so that 90% of the data points for each genome are included within the bin boundaries.

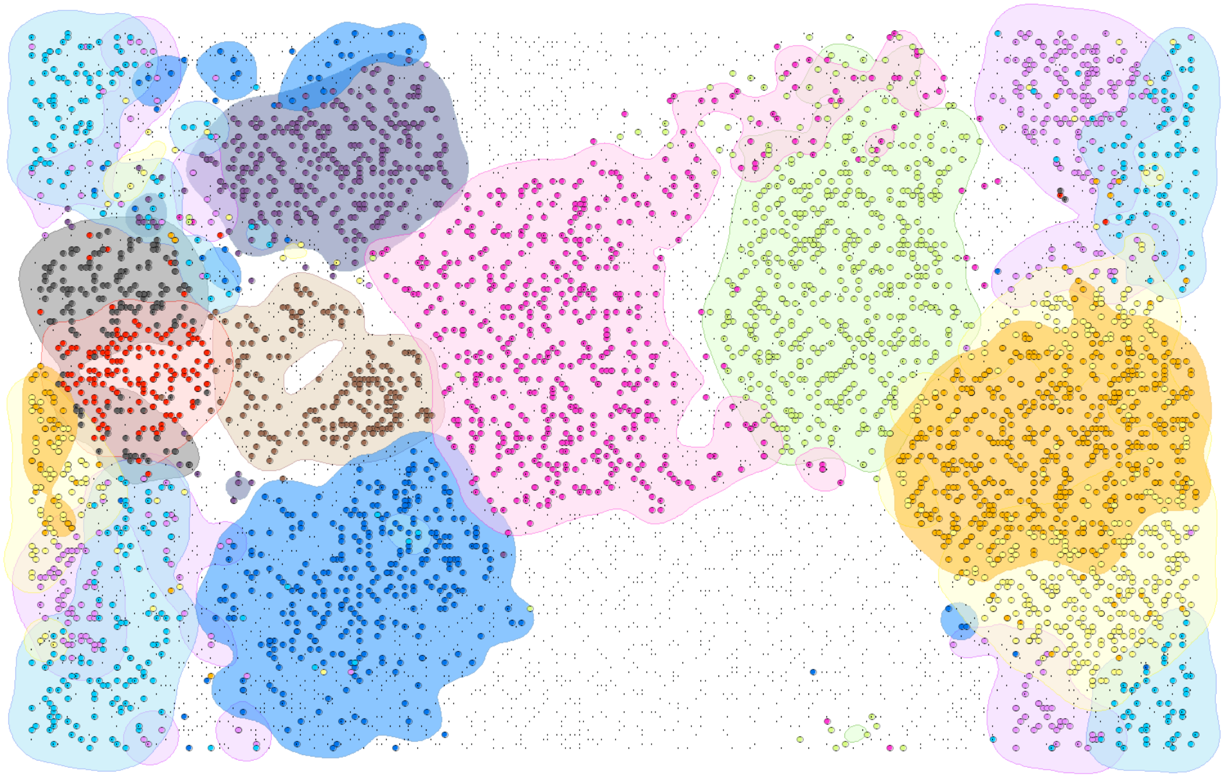

Supplement: Additional File 1 — Automated clustering of tetra-ESOM data using fixed point kernel densities. [file gb-2009-10-8-r85-S1.pdf]
