# Supplementary material for: Community-wide analysis of microbial genome sequence signatures
Source: Genome Biol. 2009 Aug 21;10(8):R85. doi: 10.1186/gb-2009-10-8-r85 (PMC2745766; doi:10.1186/gb-2009-10-8-r85)

Additional data file 2. Binning accuracy of deeply-sampled composite genomes. Bins were defined manually using background topography (the U-matrix, which is based on distance structure) as a guide. Sensitivity is the percentage of sequence fragments from each reference genome that were correctly identified; precision is the percentage of sequence fragments in each bin from the correct reference genome (ignoring unassigned fragments). (A) Accuracy of binning when all sequences are fragmented into the window sizes indicated, with the minimum fragment length considered being equal to the window size. (B) Accuracy of binning for variable fragment lengths when larger fragments are present to define the signature; 10% of each genome was randomly sampled and fragmented into the indicated size while the remaining 90% was broken into 5 kb fragments. Note that the accuracy reported is only for the smaller fragments of length indicated in the figure (i.e. 10% of the genome), and does not include the 5 kb fragments.

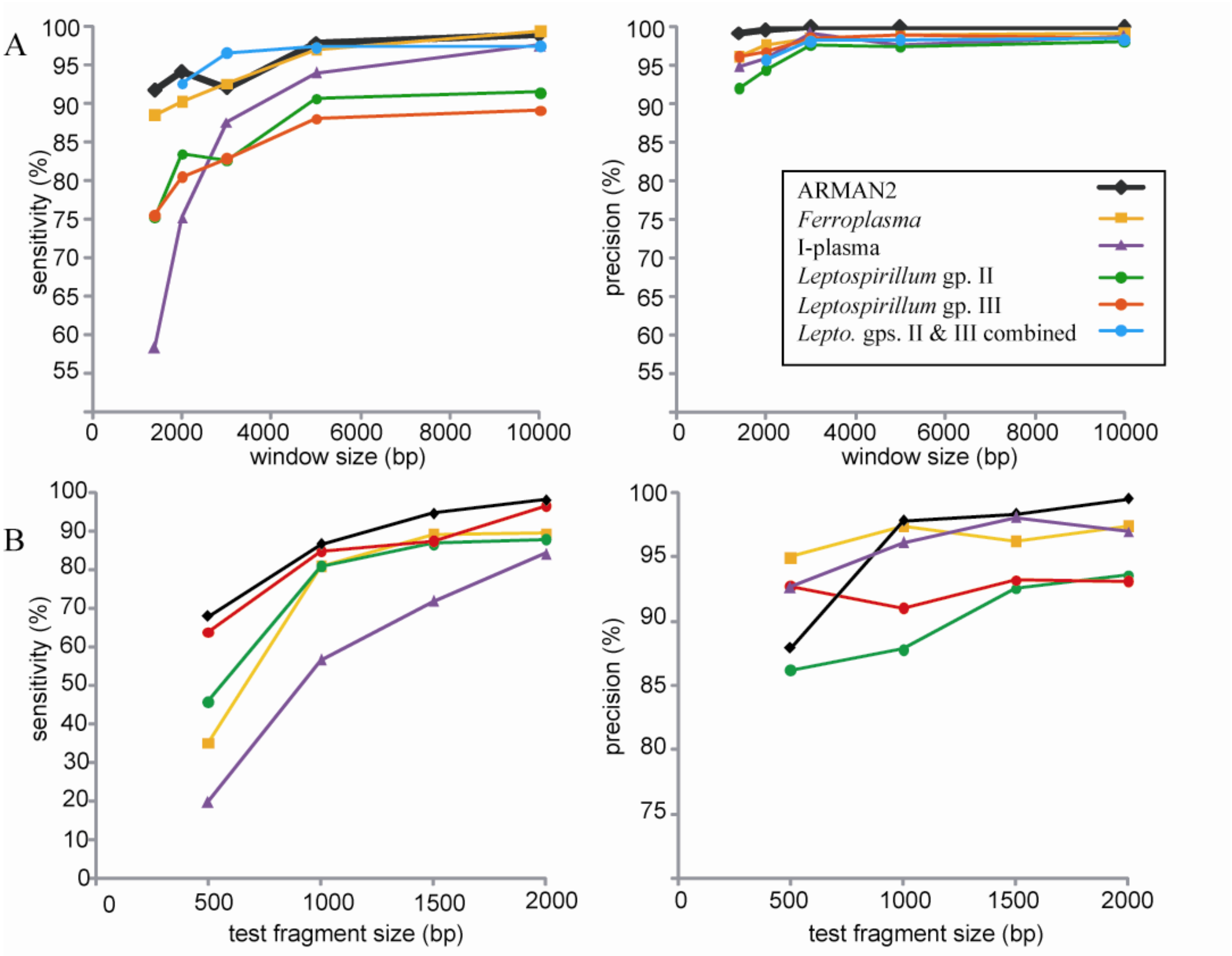

Supplement: Additional File 2 — Evaluation of binning accuracy based on deeply sampled metagenomes for which contigs are assigned to genomes with a high degree of confidence. [file gb-2009-10-8-r85-S2.pdf]
