# Supplementary material for: Community-wide analysis of microbial genome sequence signatures
Source: Genome Biol. 2009 Aug 21;10(8):R85. doi: 10.1186/gb-2009-10-8-r85 (PMC2745766; doi:10.1186/gb-2009-10-8-r85)

Additional data file 3. Binning accuracy for partial (subsampled) composite genomes. Binning and calculation of accuracy were done as described in additional file 4 with the exception that unassigned sequences were not included in the ESOM. Genomes were broken into 5-kb fragments, then this pool was randomly sampled to obtain the indicated % genome.

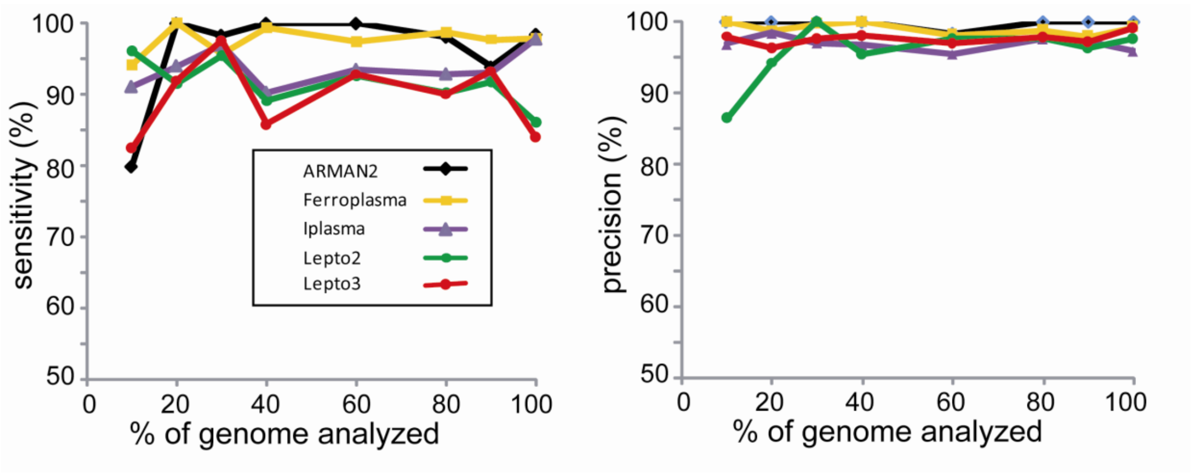

Supplement: Additional File 3 — Binning accuracy calculated for genomes that were sampled to varying extents of completeness (10 to 100%). [file gb-2009-10-8-r85-S3.pdf]
