# Supplementary material for: Community-wide analysis of microbial genome sequence signatures
Source: Genome Biol. 2009 Aug 21;10(8):R85. doi: 10.1186/gb-2009-10-8-r85 (PMC2745766; doi:10.1186/gb-2009-10-8-r85)

Additional data file 4. Average genome-wide frequency of each tetranucleotide. Tetranucleotides (columns) are sorted from left to right based on the number of G+C per tetranucleotide as indicated at top. Palindromic tetranucleotides are marked with black circles, and those that effectively distinguish closely related members of the *Thermoplasmatales* with the same %GC (E-plasma, G-plasma, *Ferroplasma* types I and II) are indicated with stars: TATA, ATAT, GATC. (A) All Iron Mountain AMD bacterial and archaeal genomes, listed from high G+C content (top) to low (bottom). (B) Archaeal and bacterial genomes for which viral genomes have been reconstructed [12]. The tetranucleotide frequencies of the hosts are shown adjacent to their viruses.

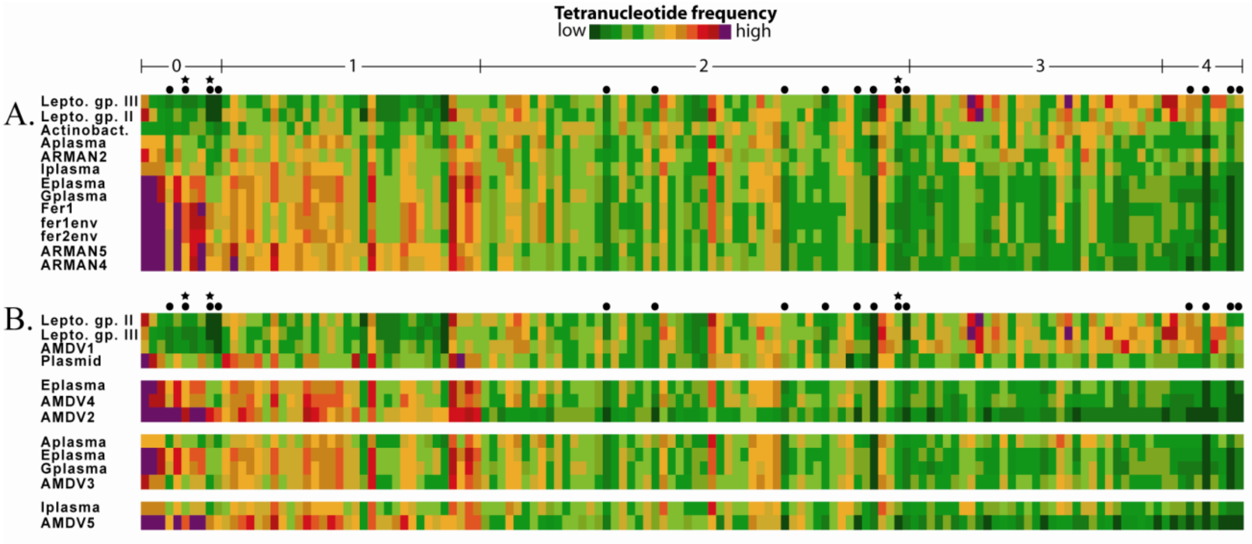

Supplement: Additional File 4 — Heat map of average genome-wide frequency of each tetranucleotide for each genome, including bacteria, archaea, viruses, and a putative plasmid. [file gb-2009-10-8-r85-S4.pdf]
