# Supplementary material for: Community-wide analysis of microbial genome sequence signatures
Source: Genome Biol. 2009 Aug 21;10(8):R85. doi: 10.1186/gb-2009-10-8-r85 (PMC2745766; doi:10.1186/gb-2009-10-8-r85)

Additional data file 5. ESOM of assembled genomes based on amino acid composition (a), codon composition (b), and tetranucleotide frequency (c). Color schemes are as in Fig. 2.

A. Amino acids:

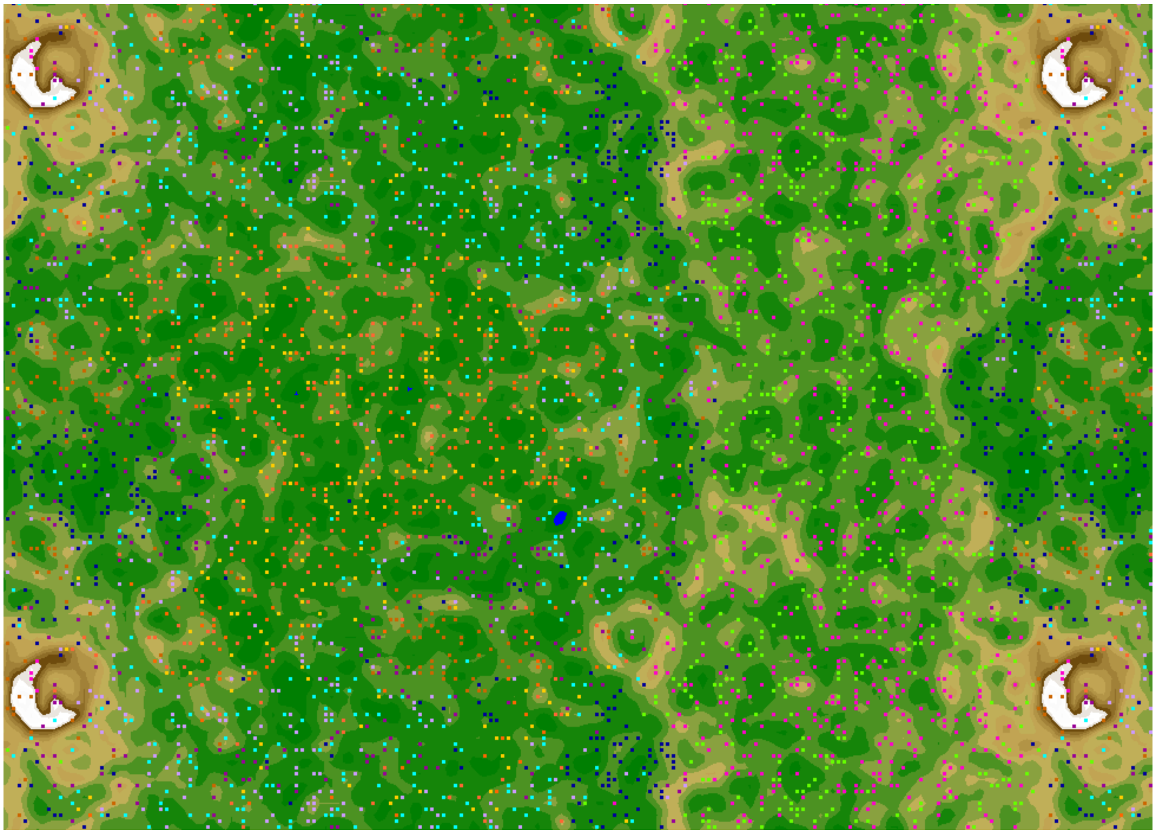

B. Codons:

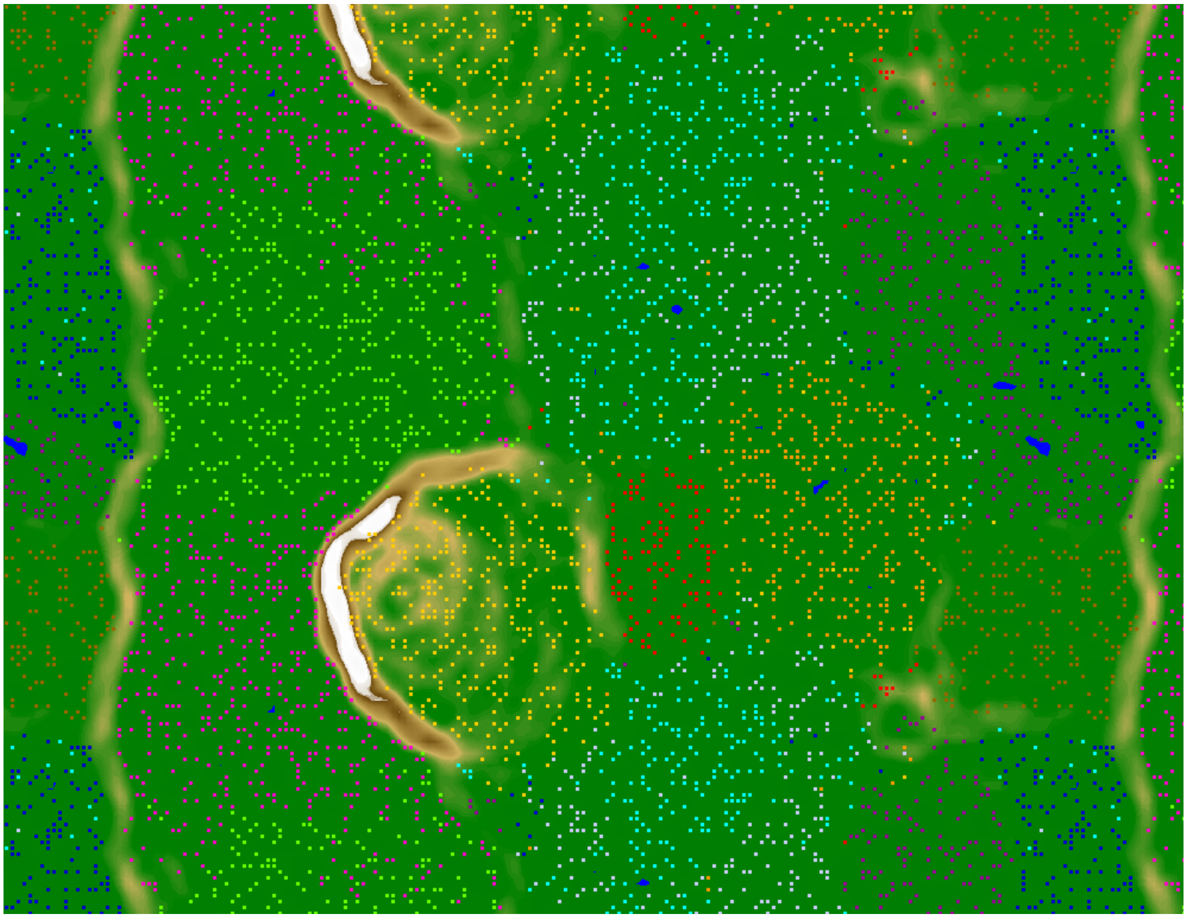

C. Tetranucleotides:

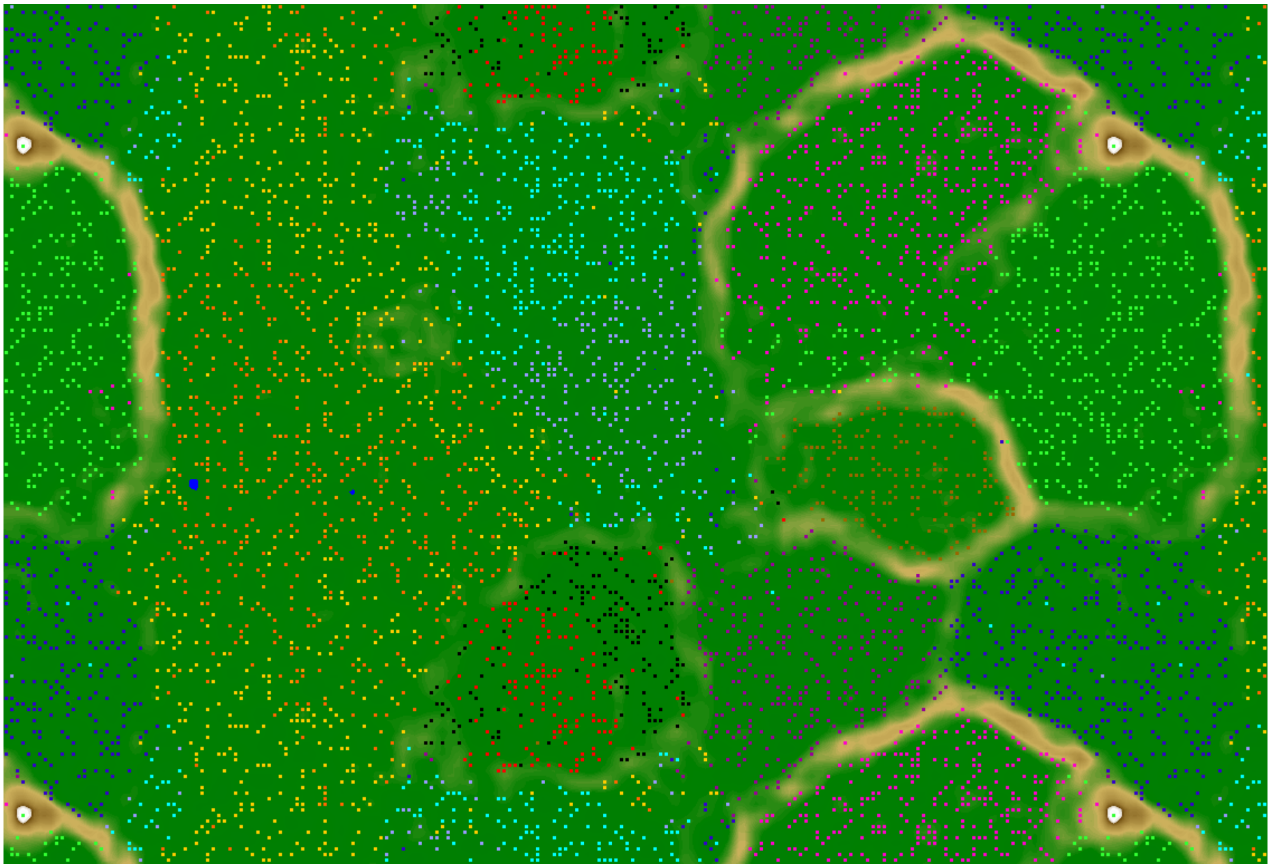

Supplement: Additional File 5 — Comparison of tetra-ESOMs of assembled genomes based on (a) amino acid composition, (b) codon composition, and (c) tetranucleotide frequency [file gb-2009-10-8-r85-S5.pdf]
