# Supplementary material for: Community-wide analysis of microbial genome sequence signatures
Source: Genome Biol. 2009 Aug 21;10(8):R85. doi: 10.1186/gb-2009-10-8-r85 (PMC2745766; doi:10.1186/gb-2009-10-8-r85)

Additional data file 6. The observed difference in frequency of each tetranucleotide between pairs of genomes correlates with the predicted difference based on codon composition. (A) A-plasma versus E-plasma; (B) Eplasma versus *Ferroplasma acidarmanus*; (C) G-plasma versus E-plasma; (D) *Leptosprillum* sp. group II versus group III.

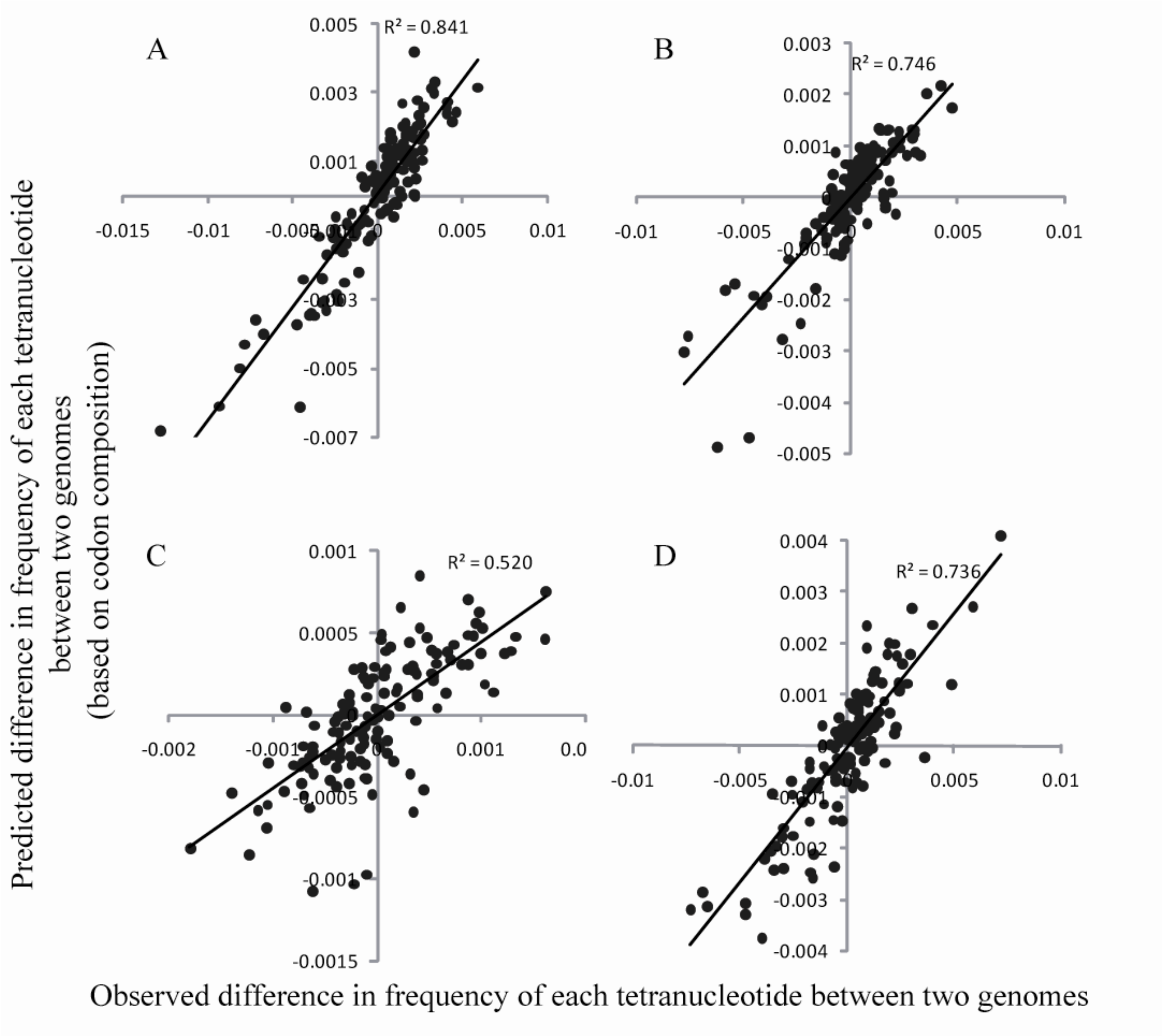

Supplement: Additional File 6 — The observed difference in frequency of each tetranucleotide between pairs of genomes correlates with the difference predicted based on codon composition. [file gb-2009-10-8-r85-S6.pdf]
