# Supplementary material for: Community-wide analysis of microbial genome sequence signatures
Source: Genome Biol. 2009 Aug 21;10(8):R85. doi: 10.1186/gb-2009-10-8-r85 (PMC2745766; doi:10.1186/gb-2009-10-8-r85)

Additional data file 7. Binning of coding and noncoding regions of assembled genomes. Noncoding regions are shown in bold with color corresponding to coding regions.

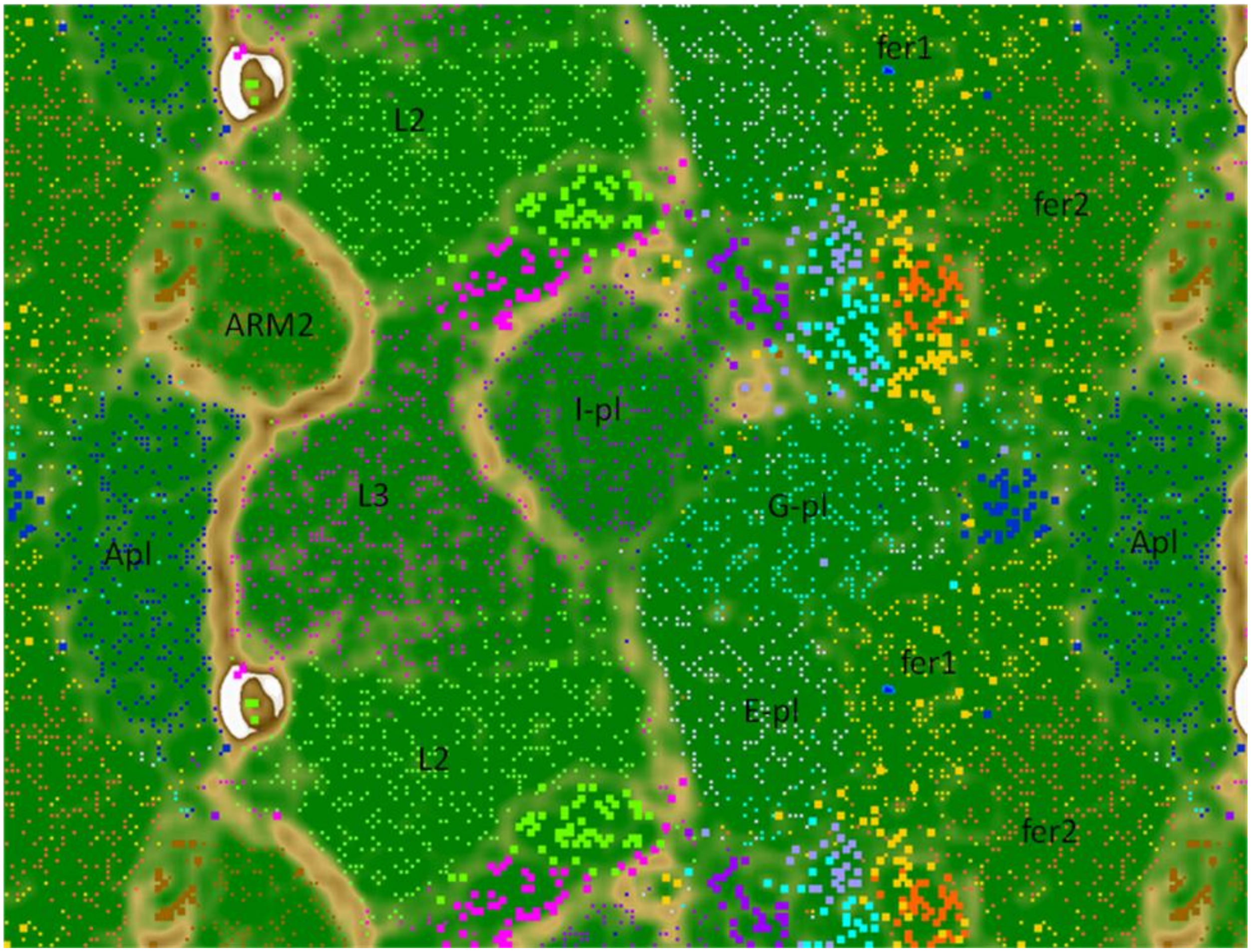

Supplement: Additional File 7 — Tetra-ESOM of deeply sampled genomes for which coding and noncoding regions were separated. [file gb-2009-10-8-r85-S7.pdf]
