# Supplementary material for: Community-wide analysis of microbial genome sequence signatures
Source: Genome Biol. 2009 Aug 21;10(8):R85. doi: 10.1186/gb-2009-10-8-r85 (PMC2745766; doi:10.1186/gb-2009-10-8-r85)
Supplement: Additional File 8 — Percentage of sequence coding for genes in comparison with the genome-wide coding percentage for incorrectly binned fragments. [file gb-2009-10-8-r85-S8.pdf]

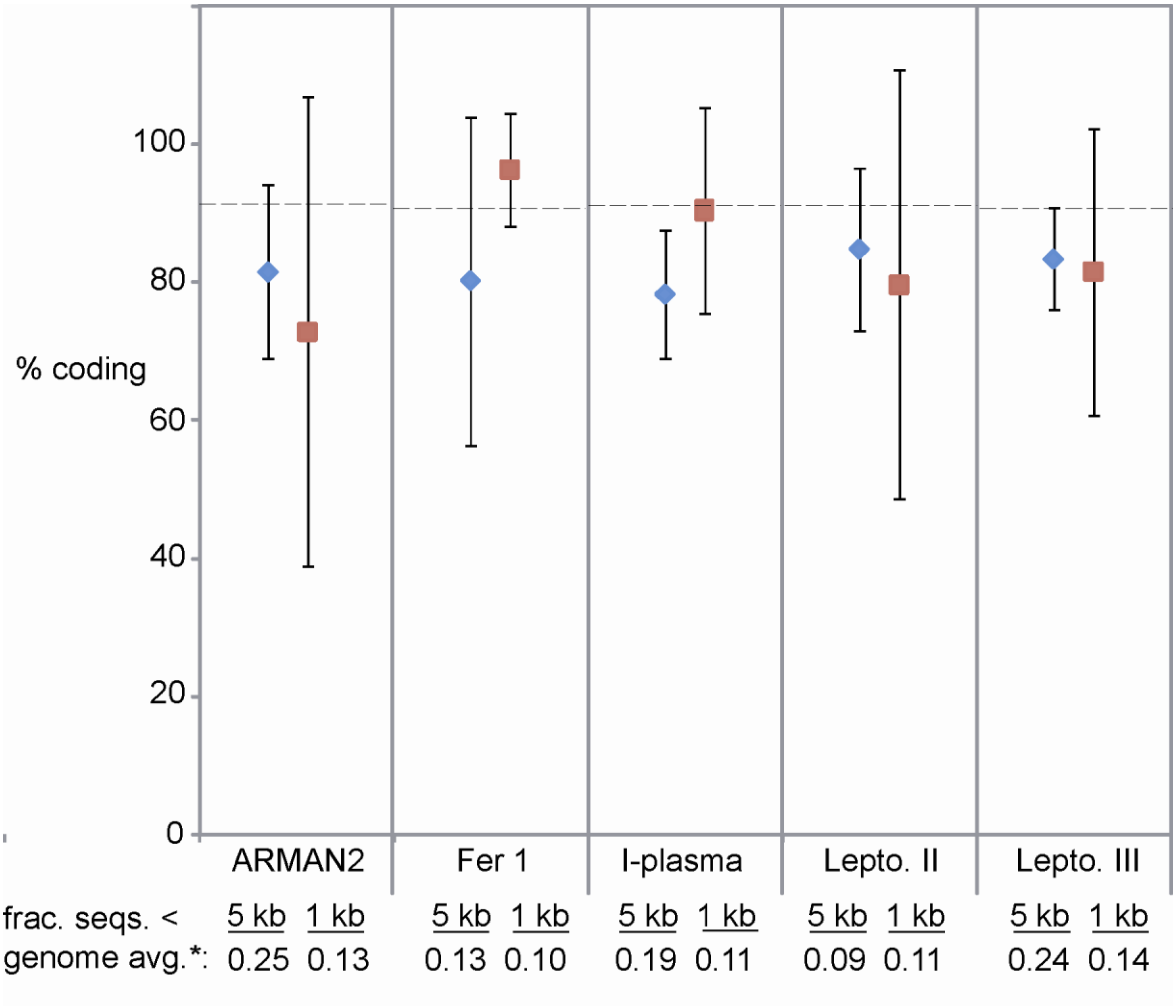

Additional data file 8. Percentage of sequence encoding genes on incorrectly binned fragments of 5 kb (blue) and 1 kb (red) length. Error bars represent one standard deviation. Dotted line indicates genome-wide average % of sequence coding for genes. * Frac. seqs. < genome avg. is the fraction of incorrectly binned sequences with coding % less than one standard deviation below the genome average.
