# Supplementary material for: Community-wide analysis of microbial genome sequence signatures
Source: Genome Biol. 2009 Aug 21;10(8):R85. doi: 10.1186/gb-2009-10-8-r85 (PMC2745766; doi:10.1186/gb-2009-10-8-r85)

Additional data file 9. ESOM with *Leptospirillum* group II genes coding for highly expressed proteins (black) or proteins enriched in the extracellular fraction (white) analyzed as separate fractions from the rest of the genome (light green). The one black data point that clusters in the *Leptospirillum* group II/III unresolved region contains the following genes:

Gene	Annotation
UBA_LeptoII_Scaffold_8692_GENE_84	Ribosomal protein L17
UBA_LeptoII_Scaffold_8692_GENE_93	Ribosomal protein L15
UBA_LeptoII_Scaffold_8692_GENE_106	Ribosomal protein L22
UBA_LeptoII_Scaffold_8692_GENE_109	Ribosomal protein L23
UBA_LeptoII_Scaffold_8692_GENE_112	Ribosomal protein S10
UBA_LeptoII_Scaffold_8692_GENE_113	Translation elongation factor Tu
UBA_LeptoII_Scaffold_8692_GENE_115	Ribosomal protein S7
UBA_LeptoII_Scaffold_8692_GENE_121	Ribosomal protein L10
UBA_LeptoII_Scaffold_8692_GENE_122	Ribosomal protein L1
UBA_LeptoII_Scaffold_8692_GENE_124	Transcription antitermination protein
UBA_LeptoII_Scaffold_8692_GENE_126	Translation elongation factor Tu
UBA_LeptoII_Scaffold_8692_GENE_142	Ribosomal protein L25

The average amino acid identity of these proteins between *Leptospirillum* groups II and III (67 +/- 14%) is not significantly different than the genome average (61 +/- 20%), therefore the difference in genome signature likely reflects unique codon usage due to translational selection.

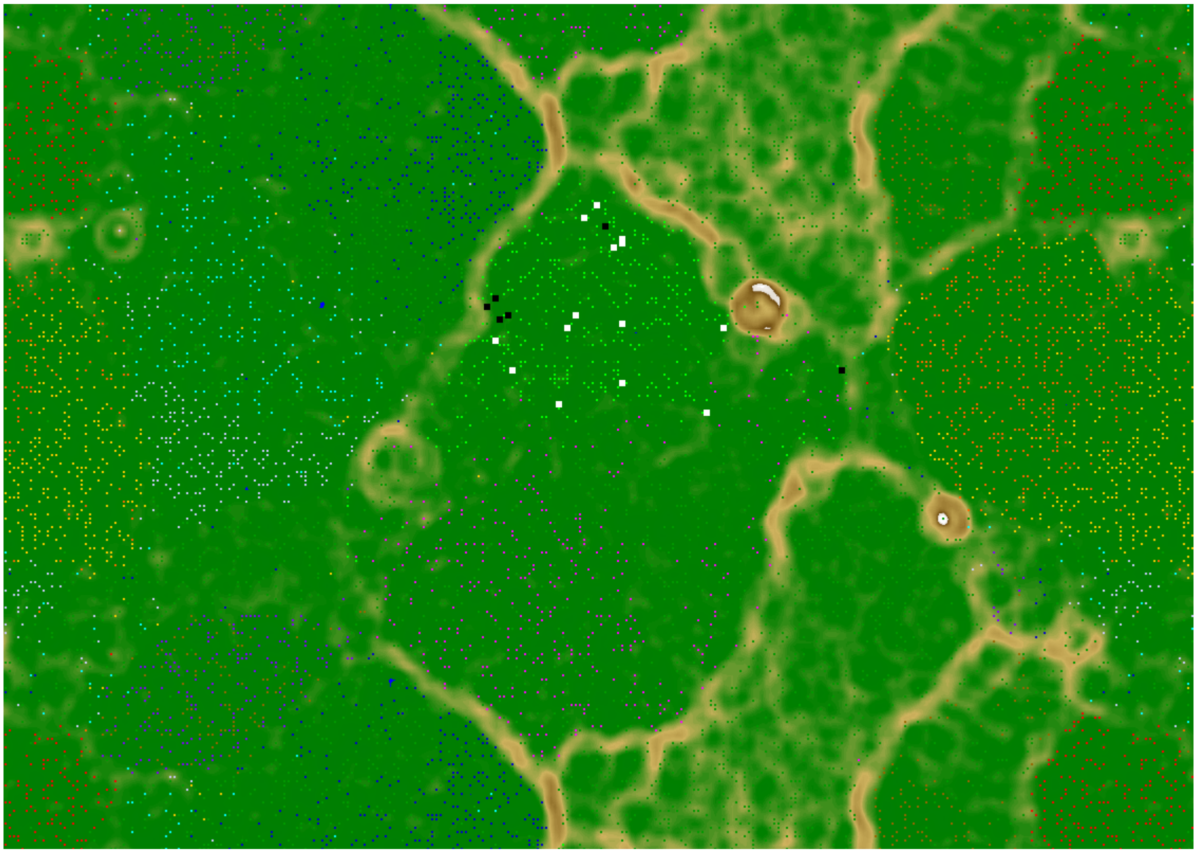

Supplement: Additional File 9 — Tetra-ESOM of Leptospirillum group II genes coding for highly expressed proteins or proteins enriched in the extracellular fraction analyzed as separate fractions from the rest of the genome. [file gb-2009-10-8-r85-S9.pdf]
