# Supplementary material for: Community-wide analysis of microbial genome sequence signatures
Source: Genome Biol. 2009 Aug 21;10(8):R85. doi: 10.1186/gb-2009-10-8-r85 (PMC2745766; doi:10.1186/gb-2009-10-8-r85)

Additional data file 10. Schematic of processes and factors influencing genome signature. Those inferred to be critical for distinguishing closely-related organisms in the AMD biofilm community are highlighted in red.

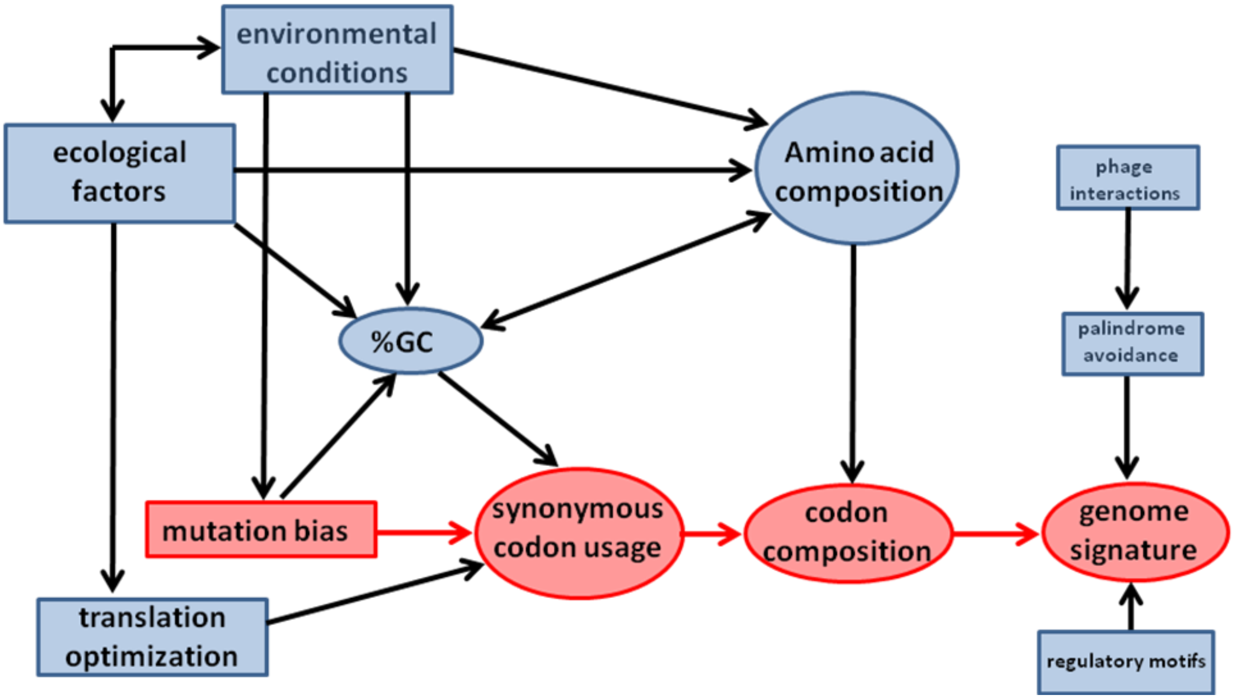

Supplement: Additional File 10 — Processes and factors influencing genome signature. [file gb-2009-10-8-r85-S10.pdf]
